# Age or health status: which influences medical insurance enrollment greater?

**DOI:** 10.7189/jogh.06.020801

**Published:** 2016-12

**Authors:** Wei Xu, Gong–Jie Cai, Guan–Nan Li, Jing–Jing Cao, Qiong–Hua Shi, Jie Bai

**Affiliations:** 1 School of International Pharmaceutical Business, China Pharmaceutical University, Nanjing, China; 2 Institute of Chinese Medical Science, University of Macau, Macau, China

## Abstract

**Background:**

The New Cooperative Medical Scheme (NCMS) for peasantries implemented in 2003 and the Urban Resident Basic Medical Insurance (URBMI) for the urban unemployed implemented in 2007 have many similarities. They both apply the financing mode of individual premiums plus government’s subsidies, and the voluntary enrollment. The Chinese government plans to integrate these two systems and build a unified basic medical insurance system for the unemployed in order to achieve the medical equity and increase the general health level. Thus, to analyze the main influencing factors of the enrollment of the urban unemployed and rural residents is very important for improving the system and securing the stability of the system during the transition.

**Methods:**

The study uses data from the China Health and Nutrition Survey (CHNS) and adopts logistic regression models to test which factors influence the enrollment of the URBMI and the NCMS under the background of rather high enrollment rate of Chinese basic medical insurances and strong fiscal support of the Chinese government, especially whether health status or age influences enrollment of these two insurances greater.

**Results:**

There is indeed some adverse selection in the URBMI and the NCMS. Whether the individual has chronic diseases have significant influence on enrollments of both the urban unemployed and rural residents, while whether the individual got ill in last four weeks just influences enrollments of the urban unemployed. Age influences enrollment greater than health status. The older the insured are, the larger the enrollment rates are.

**Conclusion:**

Because of the active support for basic medical insurances of the Chinese government, the enrollment performance of the urban unemployed and rural residents has already changed. When implementing the new policy, the government should pay attention to the willingness to enroll in and the change of enrollment performance of the insured. Therefore, under the policy of voluntary enrollment, every coordinated province and city should enlarge the proportion of young people to insuring group, optimizing the age structure, and the financing proportion of governments and individuals should be measured properly. With the increasing of governments’ subsidies, the proportion of individual’s premiums should also be increased.

The Urban Employee Basic Medical Insurance (UEBMI), the Urban Resident Basic Medical Insurance (URBMI) and the New Cooperative Medical Scheme (NCMS) compose Chinese basic medical insurance system. The UEBMI for urban employees which was built in 1998 adopts the compulsory enrollment and its premiums are contributed by both of employees and their companies, while the NCMS for rural people in 2003 and the URBMI for urban unemployed in 2007 adopt the voluntary enrollment and their funds are raised by multi–channel financing – individual contributions combining with higher governmental subsidies, which shows the great differences between the UEBMI, and the NCMS and the URBMI.

As for voluntary medical insurance, many scholars have pointed out that voluntary membership can make these schemes vulnerable to adverse selection [1-4], which may result in the opposite problem of too little coverage and thus under consumption of medical care [5,6]. Voluntary medical insurance has the potential disadvantage of not nurturing the principles of mutual support and the health care costs will increase at the same time [7]. However, some other researches show that premium subsidies for medical insurance may mitigate adverse selection and lead to excessive coverage and excessive spending on medical care [8].

The NCMS and the URBMI, using voluntary enrollment, have many similarities. Both of them have achieved a great success and exert an important effect on satisfying people’s demands for medical security and increasing people’s health level. However, with the development of social economy, the negative sides of separating these two system have appeared, like re–enrolling or re–financing. To solve these problems, the State Council of the People’s Republic of China has released A Guideline on the Integration of Basic Medical Insurance for Urban and Rural Residents which pointed that the solution should begin with the improvement of the policies and the government would propel the integration of the URBMI and the NCMS and build a unified basic medical insurance system for the unemployed in the whole country gradually [9]. This medical insurance must cover all the insured of the URBMI and the NCMS, ie, it must cover all the urban and rural residents except employees. Since there are big differences in the insured group, the financing modes and the payment modes between the URBMI and the NCMS, the unified basic medical insurance for the unemployed will face a lot of challenges. The first problem to be solved is about enrollment [10]. Only if the government instructs the residents who are eligible to insure continually for a long term, can the financing of the unified basic medical insurance be stable and sustainable.

Although the total enrollment rate of the three basic medical insurance maintains higher than 95% now, the re–enrolling rate is about 10% [11]. The actual enrollment rate of the medical insurances for the unemployed in 2014 was only 67.46%. That is to say, there some people do not enroll in any insurance, while the other people enroll in two or more medical insurances, which shows a great difference in enrollment performances of residents. Therefore, under the background of integration of the URBMI and the NCMS, because of the voluntary enrollment and a rather high enrollment rate, it is important for forecasting the structure of the insuring group and calculating the medical insurance fund to study the influencing factors of enrollment of medical insurance for the unemployed, which is also the first consideration when the government makes policies of the insurance.

Nowadays, most of scholars’ researches about influencing factors of enrollment focus on people’s health status and ability to pay [12,13]. Based on previous studies, the study holds the opinion that although the influencing factors of enrollment are various, under the background of strong aged tendency of population, the key factors are health, ability to pay, and age. Meanwhile, due to the “inclusive policies” in China, people’s premiums only take a small part of insurance financing. Therefore, the object of this study is not ability to pay but the influences of health status and age on insurance enrollment, and the comparison between them.

As for the research of the influences of health status on insurance enrollment, there are great differences between the results of them. Many studies show that the insurance market is facing serious adverse selection, especially in the insurance market of voluntary enrollment. People in poor health may have greater willingness to enroll in medical insurances [14–17]. However, other studies show that there is not a significant relationship between health status and enrollment rate. Thus there is not a severe adverse selection in insurance market [18-20]. In addition, some studies revealed that there exists a positive selection in insurance market [21,22]. Then, how health status influences insurance enrollment rate in the market of the URBMI and the NCMS is a problem that needs to be studied in the further researches.

However, as for the researches of the influences of age on insurance enrollment, the results are in agreement that people’s age influences the insurance enrollment significantly. The older the people grow the more willingness they may have to [23–25]. In addition, many research results indicate that education and marital status also have significant influences on the enrollment.

Obviously, although international and domestic scholars concentrate their research on different influencing factors of insurance enrollment, there are close connections between them. Most of these researches show that people’s health status and age have significant influences on medical insurance enrollment. Nowadays, under the background that China has gradually entered the aging society and the basic medical insurance rate is at a high level, it is meaningful to research which influencing factors can influence the enrollment better. The study is based on the voluntary enrollment policy and high enrollment rate, deeply researching whether age or health status influences medical insurance enrollment greater and further analyzing the reasons. It uses logistic regression model with the latest data of CHNS in 2011 to evaluate the influences of health status and age respectively on the enrollment of the NCMS, the URBMI, and the medical insurance for the urban and rural unemployed, then to analyze the change of enrollment performances after the expansion of the medical insurances. Finally, the study offers suggestions for the integration of the URBMI and the NCMS.

## DATA AND METHODS

Employing binary logistic regression model, the study analyzes the influencing factors of enrollment performance, especially health status and age, which influences the enrollment of the URBMI and the NCMS greater. The study tests whether it is a severe adverse selection in the URBMI and the NCMS, and with the aged tendency of population, whether age influences the enrollment greater.

The study adopts cross–sectional data of 2011 from the China Health and Nutrition Survey (CHNS), and its official website is http://www.cpc.unc.edu/projects/china. The China Health and Nutrition Survey (CHNS), an ongoing open cohort, international collaborative project between the Carolina Population Center at the University of North Carolina at Chapel Hill and the National Institute for Nutrition and Health at the Chinese Center for Disease Control and Prevention (CCDC), was designed to examine the effects of the health, nutrition, and family planning policies and programs implemented by national and local governments and to see how the social and economic transformation of Chinese society is affecting the health and nutritional status of its population. Their data from both urban and rural areas in 12 provinces and cities, which mainly cover aspects of demographics, employment status, income, demands of health services, health status, etc. are representative enough.

The number of cases which are included in the survey of medical insurance of 2011 in the CHNS is 15638. The study first eliminates individuals who enroll in the UEBMI and free health care. Then, according to the family register, the study divides registered urban and rural residents into two groups, and regards whether the individual enrolls in the URBMI and the NCMS as the dependent variables respectively. Finally, it eliminates the samples which lack main variables and gets 10 992 samples including 3761 samples of urban residents and 7231 samples of rural residents to do regression analysis. By analyzing variables, especially health–related variables and age, and studying whether these variables influence enrollment significantly, the study investigates the enrollment mechanism of the inclusive URBMI and NCMS.

### Variables

The enrollment of the URBMI and the NCMS can be influenced by many factors. Considering data from the CHNS, the research objective and research needs, the study decides whether the individual enrolls in the medical insurance for the unemployed – whether the urban residents who do not enroll in the UEBMI and free health care enroll in the URBMI, whether the rural residents enroll in the NCMS or whether residents enroll in one of these two insurances–––to be the dependent variable. The dependent variable is a binary variable, which means that it has two values, enrolling as 1, not enrolling as 0. There totally 10 992 people are analyzed. The amount of people who enroll in the insurances is 8902, which shows the enrollment rate of the URBMI and the NCMS in 2011 was 81.0%, not a high number. Among them, the amount of urban residents is 3761 and there are 2291 people covered by the URBMI, leading to the enrollment rate of 60.9%; the amount of rural residents is 7231 and there are 6611 people covered by the NCMS, getting the enrollment rate of 91.4%. Generally speaking, the enrollment rates of the URBMI and the NCMS are rather different. Therefore, it is important for improving the basic medical insurance for the unemployed based on the voluntary enrollment to compare the enrollment performances of people enrolling in these two insurances and analyze the main causes of the differences.

The independent variables are classified into three categories: health consciousness and health status, controlled variables, and age. The variables in health consciousness and health status include whether the individual received health care in last four weeks, whether the individual got ill in last four weeks and whether the individual has chronic diseases. The controlled variables includes education, per capita income of their family for a year, whether the individual is on the jobs, living districts, gender, and nationality.

The health consciousness is measured by whether the individual received health care in last four weeks. It is acknowledged that people with higher health consciousness are more likely to avoid risk and choose to enroll in medical insurances. However, in the data of the CHNS, there is no direct indicator for preferences in risk avoiding. Referring to the conventional practices in international empirical literatures of health economics [26], the study uses individual health behaviors to reflect their preferences in risk avoiding, which means that people who want to avoid risk will pay attention to health care and purchase medical insurances. Finally, it chooses whether the individual received health care (such as physical examination, visual examination, blood test, test of hypertension, cancer screening, etc.) in last four weeks as the indicator.

The study regards whether the individual got ill in last four weeks and whether the individual has chronic diseases as the indicators of health status and predicts the expected size of medical needs by actual and hidden health problems. In the field of medical insurance, the adverse selection is shown as people with poor health status are more willing to enroll in the insurances, ie, their enrollment rate is higher. Because it was hard to know their health status and medical needs when respondents chose enrollment performances in the survey of CHNS, the study considers whether the individual got ill in last four weeks as the expected medical needs and whether the individual has chronic diseases as the actual medical needs. As for whether the individual has chronic diseases, according to the questionnaires, if an individual has been diagnosed as any one or more of hypertension, diabetes, myocardial infarction, stroke, neoplasm, fracture and asthma, he will be considered to have chronic diseases.

Among the controlled variables, the income is divided based on the quartile of the per capita income of their family for a year. Since the income of urban residents and of rural residents have a big difference and the individual financing levels of the URBMI and of the NCMS are also different, the study divides the per capita income of their family for a year of urban residents and of rural residents into quartiles and does regression analyses respectively. As for district, the Jiangsu Province, the Shandong Province, the Beijing City and the Shanghai City are classified as the East; the Henan Province, the Hubei Province and the Hunan Province are classified as the Middle; the Guangxi Zhuang Autonomous Region, the Guizhou Province and the Chongqing City are classified as the West; the Liaoning Province and the Heilongjiang Province are classified as the Northeast.

The evaluation method of the variables and descriptive statistical results are shown in **Table 1**.

**Table 1:** The descriptive statistics of enrollment of the medical insurances for the urban unemployed and rural residents

Independent variables and definition	The urban unemployed			The rural residents			Merging			
Rate (%)	Mean	Standard deviation	Rate (%)	Mean	Standard deviation	Rate (%)	Mean		Standard deviation
**Receiving health care in last four weeks:**										
Yes = 1	9.40	0.094	0.292	5.10	0.051	0.221	6.60	0.066	0.248	
No = 0	90.60			94.90			93.40			
**Having chronic diseases:**										
Yes = 1	20.80	0.208	0.406	16.00	0.16	0.366	17.60	0.176	0.381	
No = 0	79.20			84.00			82.40			
**Getting ill or injured in last four weeks:**										
Yes = 1	19.50	0.195	0.396	14.60	0.146	0.354	16.30	0.163	0.369	
No = 0	80.50			85.40			83.70			
On the job:										
Yes = 1	27.80	0.278	0.448	54.60	0.546	0.498	45.40	0.454	0.498	
No = 0	72.20			45.40			54.60			
**Education:**										
Elementary school or below = 1	43.20	1.637	0.607	60.10	1.409	0.512	54.30	1.487	0.557	
Middle school or high school = 2	49.90			38.90			42.70			
College or above = 3	6.90			1.00			3.00			
**Income:**										
Low–income = 1	25.00	2.499	1.118	25.00	2.499	1.118	25.00	2.499	1.118	
Lower–middle–income = 2	25.10			25.00			25.00			
Upper–middle–income = 3	24.90			25.00			25.00			
High–income = 4	25.00			25.00			25.00			
**Gender:**										
Male = 1	44.70	1.553	0.497	46.70	1.533	0.499	46.00	1.54	0.498	
Female = 2	55.30			53.30			54.00			
**Nationality:**										
Han nationality = 1	93.30	0.933	0.251	85.70	0.857	0.35	88.30	0.883	0.322	
National minority = 0	6.70			14.30			11.70			
**District:**										
East = 1	47.50	1.944	1.029	19.70	2.48	0.985	29.20	2.297	1.032	
Middle = 2	18.90			28.90			25.40			
West = 3	25.30			35.10			31.70			
Northeast = 4	8.30			16.30			13.60			
**Age:**										
0–17.99 = 1	26.40	4.036	2.226	19.00	4.277	2.035	21.60	4.194	2.105	
18–24.99 = 2	4.70			4.20			4.30			
25–34.99 = 3	7.70			7.80			7.80			
35–44.99 = 4	11.80			16.20			14.70			
45–54.99 = 5	15.40			18.80			17.60			
55–64.99 = 6	18.30			19.70			19.20			
65– = 7	15.90			14.20			14.80			

On the basis of characteristics of the URBMI and the NCMS, any urban residents without formal employment, including children, the elderly, and other unemployed urban residents, can enroll in the URBMI, while the rural residents, taking family as a unit, can enroll in the NCMS voluntarily. When doing a further analysis, among the group covered by the URBMI, 26.4% of them are minors; 34.2% of them being older than 55, 72.2% of them having no jobs; and for rural residents, the limits of natural resources and economic conditions restraint their medical needs to some degree. Most of these residents are the vulnerable group and their health should be secured by medical insurances.

In the social medical insurance system, the relative departments of the government will not investigate individuals’ health status like commercial insurance companies. Therefore, the health information of the insured cannot be observed by the insurers, which exacerbates the information asymmetry. Under the policy of voluntary enrollment, if the individuals having different health risks enjoy the same insurance price, when the insurer offers insurances with different security levels, the individuals having higher health risk will choose to enroll in the insurance with higher security level (with low out–of–pocket). Because both the URBMI and the NCMS are designed as enrolling voluntarily and are of single security mode, if there are severe adverse selections in these two medical insurances, the results of the logistic regression analyses will perform as the enrollment rate of the high–risk individuals are higher and whether the individuals enroll in the insurance is related with the level of risk significantly. If age influences enrollment greater than health status, then under the condition of other fixed variables, when put age into the model, the coefficients of health status will decrease much, and the influences of health status on enrollment will not be significant even.

## RESULTS

The study does a simple cross–tabulation analysis of the independent variables as whether the individual got ill in last four weeks, whether the individual has chronic diseases, whether the individual received health care in last four weeks and age. The results are shown in **Table 2**. Finally, adopting the binary logistic regression model, it takes whether the individual enrolls in the medical insurances as the dependent variable and put the independent variables into the logistic analysis. The results are shown in **Table 3** and **Table 4**.

**Table 2:** The cross-tabulation analysis of health consciousness, health status and enrollment

Health consciousness and health status	URBMI			NCMS			Merging		
Number of people	Number not enrolling (%)	Number enrolling (%)	Number of people	Number not enrolling (%)	Number enrolling (%)	Number of people	Number not enrolling (%)	Number enrolling (%)
Getting ill or injured in last four weeks	734	252 (34.3)	482 (65.7)	1059	89 (8.4)	970 (91.6)	1793	190 (10.6)	1603 (89.4)
Not getting ill or injured in last four weeks	3027	1218 (40.2)	1809 (59.8)	6172	531 (8.6)	5641 (91.4)	9199	1152 (12.5)	8047 (87.5)
Having chronic diseases	781	254 (32.5)	527 (67.5)	1155	83 (7.2)	1072 (92.8)	1936	187 (9.7)	1749 (90.3)
Not having chronic diseases	2980	1216 (40.8)	1764 (59.2)	6076	537 (8.8)	5539 (91.2)	9056	1155 (12.8)	7901 (87.2)
Receiving health care in last four weeks	354	125 (35.3)	229 (64.7)	372	47 (12.6)	325 (87.4)	726	102 (14.0)	624 (86.0)
Not receiving health care in last four weeks	3407	1345 (39.5)	2062 (60.5)	6859	573 (8.4)	6286 (91.6)	10 266	1240 (12.1)	9026 (87.9)
Older than 55	1286	380 (29.5)	906 (70.5)	2455	129 (5.3)	2326 (94.7)	3741	253 (6.8)	3488 (93.2)
Younger than 55	2475	1090 (44.0)	1385 (56.0)	4776	491 (10.3)	4285 (89.7)	7251	1089 (15.0)	6162 (85.0)

URBMI – Urban Resident Basic Medical Insurance; NCMS – New Cooperative Medical Scheme

**Table 3:** The results of logistic regression analysis of influences of whether the individual got ill or injured in last four weeks on enrollment

Independent variables and definition	URBMI			NCMS			Merging			
Model I	Model II	Model III	Model I	Model II	Model III	Model I		Model II	Model III
**Receiving health care in last four weeks (no = 0):**										
Yes = 1	1.189	0.377	0.195	–0.462‡	–0.34†	–0.362†	–0.216*	0.539		0.159
**Getting ill or injured in last four weeks (no = 0):**										
Yes = 1	0.253‡	0.265‡	2.503	0.316	0.603	0.356	0.211†	0.324‡		2.107
**On the job (no = 0):**										
Yes = 1		–0.231‡	–0.197†		1.103‡	0.786‡		1.026‡		0.771‡
**Education (elementary school or below = 1):**										
Middle school or high school = 2		0.303‡	0.356‡		–0.11	–0.098		–0.23‡		–0.312‡
College or above = 3		–0.065	0.243		–1.034‡	–0.895‡		–1.265‡		–1.246‡
**Income (low–income = 1):**										
Lower–middle–income = 2		0.245†	0.248†		0.221*	0.248*		0.882		1.114
Upper–middle–income = 3		0.431‡	0.429‡		–0.009	0.035		1.567		0.8
Upper–income = 4		0.613‡	0.567‡		–0.247†	–0.232*		1.432		0.791
**Gender (male = 1):**										
Female = 2		0.124*	2.523		0.146*	0.043		0.146†		0.724
**Nationality (national minority = 0):**										
Han nationality = 1		0.366	0.202		–0.586‡	–0.601‡		–0.653‡		–0.67‡
**District (east = 1):**										
Middle = 2		–0.22†	–0.24†		0.188	0.196		0.329‡		0.314‡
West = 3		–0.794‡	–0.795‡		0.487‡	0.605‡		–0.013		0.075
Northeast = 4		–0.536‡	–0.63‡		–0.098	–0.082		–0.176*		–0.209†
**Age (0–17.99 = 1):**										
18–24.99 = 2			–0.47†			0.612‡				0.425‡
25–34.99 = 3			–0.327*			0.197				0.238*
35–44.99 = 4			0.102			0.665‡				0.584‡
45–54.99 = 5			0.413‡			0.883‡				0.95‡
55–64.99 = 6			0.573‡			1.131‡				1.394‡
65– = 7			0.75‡			1.089‡				1.108‡
Constant	0.396‡	0.231†	0.078	2.395‡	2.224‡	1.79‡	1.956‡	2.16‡		1.803‡

URBMI – Urban Resident Basic Medical Insurance; NCMS – New Cooperative Medical Scheme

*Significant at 90% confidence level.

†Significant at the 95% confidence level.

‡Significant at the 99% confidence level.

**Table 4:** The result of logistic regression analysis of influences of whether the individual has chronic diseases on enrollment

Independent variables and definition	URBMI			NCMS			Merging		
Model I	Model II	Model III	Model I	Model II	Model III	Model I	Model II	Model III
**Receiving health care in last four weeks (no = 0):**									
Yes = 1	1.406	0.143	0.195	–0.5‡	–0.392†	–0.362†	–0.218*	0.41	0.078
**Having chronic diseases (no = 0):**									
Yes = 1	0.358‡	0.254‡	2.278	0.259†	0.37‡	0.67	0.327‡	0.43‡	0.181*
**On the job (no = 0):**									
Yes = 1		–0.234‡	–0.197†		1.093‡	0.786‡		1.025‡	0.762‡
**Education (elementary school or below = 1):**									
Middle school or high school = 2		0.291‡	0.356‡		–0.104	–0.098		–0.237‡	–0.307‡
College or above = 3		–0.071	0.243		–1.025‡	–0.895‡		–1.275‡	–1.238‡
**Income (low–income = 1):**									
Lower–middle–income = 2		0.228†	0.248†		0.234*	0.248*		1.085	1.014
Upper–middle–income = 3		0.416‡	0.429‡		–0.003	0.035		1.585	0.811
Upper–income = 4		0.592‡	0.567‡		–0.24†	–0.232*		1.044	0.832
**Gender (male = 1):**									
Female = 2		0.126*	2.523		2.547	0.043		0.149**	0.653
**Nationality (national minority = 0):**									
Han nationality = 1		0.536	0.202		–0.592‡	–0.601‡		–0.645‡	–0.666‡
**District (east = 1):**									
Middle = 2		–0.219†	–0.24†		0.214*	0.196		0.346‡	0.303‡
West = 3		–0.779‡	–0.795‡		0.517‡	0.605‡		0.031	0.065
Northeast = 4		–0.56‡	–0.63‡		–0.086	–0.082		–0.188*	–0.214†
**Age (0–17.99 = 1):**									
18–24.99 = 2			–0.47†			0.612‡			0.432‡
25–34.99 = 3			–0.327*			0.197			0.247*
35–44.99 = 4			0.102			0.665‡			0.605‡
45–54.99 = 5			0.413‡			0.883‡			0.99‡
55–64.99 = 6			0.573‡			1.131‡			1.459‡
65– = 7			0.75‡			1.089‡			1.193‡
Constant	0.372‡	0.248†	0.078	2.359‡	2.232‡	1.79‡	1.936‡	2.121‡	1.808‡

URBMI – Urban Resident Basic Medical Insurance; NCMS – New Cooperative Medical Scheme

*Significant at the 90% confidence level.

†Significant at the 95% confidence level.

‡Significant at the 99% confidence level.

### Cross–tabulation analysis of health status and age, and enrollment

To reflect the relationship between whether the individual enrolls in the URBMI as well as the NCMS and health status as well as age, **Table 2** shows the cross–tabulation analysis of health consciousness, health status, age and whether the individual enrolls in the medical insurances.

It can be seen from **Table 2** that the enrollment rates of people with different health consciousness, different health status and different ages are distinct. As for the URBMI which has a rather low enrollment rate, the enrollment rate of people who got ill in last four weeks, have chronic diseases, received health care in last four weeks or are older than 55 are higher than it of people who did not and are not; as for the NCMS which has a high enrollment rate, the enrollment rate of people with poor health status and it of good health status have little differences, but people older than 55 are more willing to enroll in the insurance. Merging data of the URBMI and the NCMS, the difference is smaller than the data only of the URBMI. What should be paid attention to is that the data in **Table 2** also report that whether received health care in last four weeks, as the variables reflecting health consciousness, influences enrollment rate slighter than health status, while age influences enrollment rate greater than health status.

The results of cross–tabulation analysis in **Table 2** only explain that there are differences in enrollment rates of people with different health consciousness, health status and ages from the perspective of percentage. It need further analysis by logistic regression model to explore whether there is connection between enrollment and health status, health consciousness and age, how strong the connection is and which variable influences the insurance enrollment greater.

### Influences of whether the individual got ill in the last four weeks on insurance enrollment

In the logistic regression model, the likelihood–ratio is applied to test and backward stepwise manner is applied to filter variables. If the variable's significance level is smaller than 0.05, it will be analyzed continually, but if its significance level is larger than 0.10, it will be excluded. The influences of whether the individual got ill in last four weeks on insurance enrollment are shown in **Table 3**. Model I only estimates influences of whether the individual got ill in last four weeks and whether the individual received health care on insurance enrollment and broadens the presupposition of the model gradually. Model II adds controlled variables that are whether the individual is on the job, education, income, gender, nationality, district, etc. Model III adds the variable of age on the basis of Model II.

It can be seen from Model I and Model II in **Table 3** that as for people insuring the URBMI and merging people insuring the URBMI and the NCMS, not considering other independent variables, whether the individual got ill in last four weeks influences insurance enrollment significantly. In addition, the enrollment rate of people who got ill in last four weeks is higher than it of people who did not get ill in last four weeks, while whether the individual got ill in last four weeks does not influence people just involving in the NCMS much.

The regression results of Model III show that after adding the variable of age, whether the individual got ill in last four weeks, which has influenced insurance enrollment significantly in Model I and Model II, does not influence it much yet. That is to say, age influences individuals on enrollment greater than whether the individual got ill in last four weeks.

### Influences of whether the individual has chronic diseases on insurance enrollment

Using the same method, the results of influences of whether the individual has chronic diseases on insurance enrollment are shown in **Table 4**. Model I estimates influences of whether the individual has chronic diseases and whether the individual received health care in last four weeks on insurance enrollment. The variables put into Model II and Model III are totally the same as the variables in last part.

Model I and Model II in **Table 4** show that whether the individual has chronic diseases influences whether the individual enrolls in the insurances significantly. People with chronic diseases are more willing to enroll in the insurances than people without them.

Similar to the situation happened to whether the individual got ill in last four weeks, when adding the variable of age, the previous significant variable in Model I and Model II – whether the individual has chronic diseases – are not significant anymore. However, regarding merging residents, whether the individual has chronic diseases still has a statistically significant influence at the 10% level. Age influences enrollment greater than the individual’s actual health status (whether the individual has chronic diseases) toward urban unemployed and rural residents.

### Summary

Analyzing **Table 3** and **Table 4**, the results show that the adverse selection exists in the URBMI and the NCMS. Compared with the adverse selection in the NCMS, the adverse selection in the URBMI is more evident but not severe. Whether the individual got ill in last four weeks and whether the individual has chronic diseases influence the enrollment of the URBMI significantly and people having gotten ill in last four weeks or having chronic diseases are more willing to enroll in the insurance; whether the individual got ill in last four weeks has no significant influence on the NCMS, but whether the individual has chronic diseases has, which is performed as the enrollment rate of rural residents who have chronic diseases is higher than those who do not have.

What should be noticed is that when we put the variable of age in the analysis, the influences of whether the individual got ill in last four weeks on the enrollment rates of urban residents, rural residents or urban and rural residents together are not significant, and the influences of whether the individual has chronic diseases on the enrollment rates of urban residents and rural residents are also not significant, but the level of influences on the enrollment rates of urban and rural residents together is still in 10% which is significant. Whether to take actual health status or to take expected health status as the variable indicating health status, the influences of age on insurance enrollment are similar and are greater than the influences of health status. In another word, in the market of the URBMI and the NCMS, there are not severe adverse selection. In addition, the results of **Table 3** and **Table 4** show that the enrollment rate will increase with age growing.

Above all the samples in this study, 20% of urban samples and 13% of rural samples were students, in addition, some samples were preschool children. For these samples, their enrolment decisions were made by their parents, thus it may have some influences on the final results and conclusion. Although this study cannot reveal the influences of parents on the enrolment willingness of these samples, it verified that age and health status were the main reasons that influence students and preschool children’s enrolment willingness.

## DISCUSION

### Easing adverse selection and changing enrollment performances of residents by inclusive policies

According to the data from the CHNS of 2011, under the policy of voluntary enrollment, the URBMI and the NCMS in China cover a great range of people. Though there are problems of adverse selection in these two basic medical insurance markets, they are not severe. Age influences insurance enrollment greater than health status (whether the individual got ill in last four weeks and whether the individual has chronic diseases). The older the group are, the higher the enrollment rate is. The study considers that it is related to the inclusive design of the system for one thing, and changes of enrollment performances of the urban unemployed and rural residents for another thing.

In the market of the URBMI and the NCMS, the central government offers large subsidies. After the start of the new health–care reform in 2009, governments at all levels committed 850 billion yuan to medical field in the first three years, mainly to support five reforms including construction of basic medical security system. As for the construction of medical security system for the whole population, the government has spent over 680 billion from 2009 to 2012 to subsidy the URBMI and the NCMS. The increase of fiscal subsidy per capita in the URBMI are always larger than the increase of individual premiums, leading to a higher proportion of government subsidies to financing per capita, from 60.8% in 2009 to 79.3% in 2014. The ratio of government subsidies to individual premiums is close to 4:1, which means that the actual individual financial burdens are not heavy. The massive fiscal supports stimulate people’s willingness to enroll in the medical insurances and develop the coverage of the insurances effectively, which may be the reason why there is no severe adverse selection in the market of basic medical insurances in China.

Analyzing **Table 1** and **Table 2**, at present, the self–care consciousness of the urban unemployed and rural residents is still very low. The percentage of the urban unemployed who received health care in last four weeks is only 9.4%, while it of rural residents is only 5.1%. The percentage of individuals who received health care in last four weeks is similar to the percentage of individuals who enroll in the health care. Because of weak self–care consciousness, when the urban unemployed and rural residents decide whether they insure or not, their health status and medical needs will not influence their decisions. They pay more attention to their ages and potential health risks. In the process of developing basic medical insurances in China, under the background of large financial subsidies from Chinese government and high general enrollment rates of the insurances for the urban unemployed and rural residents, the enrollment performances of them may change. With age growing, the potential health risks and medical needs increase and the urban unemployed and rural residents are more sensitive to age. Therefore, they choose to enroll in the medical insurances.

### Optimizing the structure of insuring group and increasing anti–risk ability of medical insurance funds

The results of the study show that although age influences insurance enrollment greater than health status, the enrollment performance that the enrollment rates increase with age growing will go against optimizing the structure of insuring group. With the growing aged tendency of population, the general enrollment rate of Chinese basic medical insurances for the urban unemployed and rural residents can maintain stable or even higher, but it also results in an unreasonable structure of insuring group. If the reimbursement level of these insurances raises continually, the burden of the medical insurance funds must be increased, which brings about a danger to them. To optimize the structure of insuring group, there must more young adults be appealed to enroll in the insurances, and then, the basic medical insurances exert the effect of social assisting and risk sharing under the law of great number.

Learning from the practices in China, the great input of government cannot keep the sustainability of the basic medical insurances. After the new reform of medical system, medical expenditures in our country increase rapidly, causing the growth rate of expenditures of medical insurance funds higher than the growth rate of income of it. In some provinces, the income of their medical insurance funds cannot pay out the cost of the funds and the insurance funds are dangerous. To keep the insurance funds from risks, the Ministry of Human Resources and Social Security of the People’s Republic of China and the Ministry of Finance of the People’s Republic of China has investigated in 2009 and suggested that only the fund can satisfy the financial demand of six to nine month, can the medical insurances be well operated. The data from the Ministry of Human Resources and Social Security of the People’s Republic of China show that compared with it of 2013, the length of payable months of 2014 in 22 provinces become shorter. There are six provinces that the length of payable months is shorter than six months. The medical insurance funds in many regions are in danger and the sustainability of social medical insurances face a great challenge. In addition, the policies of the insurance that integrate s the URBMI and the NCMS for urban unemployed and rural residents will follow the principle: the financing standard should be the lower one; the reimbursement level should be the higher one; the range of pharmaceuticals should be the larger one, which increases the expenditures of the medical insurance funds [27]. Therefore, it should keep the fund resources stable, optimize the financing structure, enlarge the coverage of the insurance, enlarge the medical insurance fund, and increase the anti–risk ability of the fund.

## CONCLUSION

The results of the study show that although both of the URBMI and the NCMS apply the policy of voluntary enrollment, the problems of adverse selection in them are not severe. Age influences people’s enrollment of medical insurances. The older the insuring group are, the higher their enrollment rate is. This enrollment performance is just like adverse selection which goes against the sustainability of the medical insurances.

Analyzing from the perspective of actuarial studies, when people with different ages and different health status enroll in the medical insurance simultaneously and continually, the security function of insurance fund can be exerted and the insurance fund can be operated sustainably. However, based on the analyses above, it can be known that the older the individual is, the greater the willingness to enroll in the insurance is and also the higher the enrollment rate is. With the growing aged tendency of population, this will be more and more evident. Therefore, under the background of integrating the URBMI and the NCMS together, every provinces and cities should increase the proportion of young adults in the market of medical insurance and optimize the structure of insuring group. Moreover, the financing of government and individual should be divided properly. When the government’s subsidies increase, the proportion of individual’s premiums should also be raised.
